# Data in support of genetic architecture of glucosinolate variations in *Brassica napus*

**DOI:** 10.1016/j.dib.2019.104402

**Published:** 2019-08-14

**Authors:** Varanya Kittipol, Zhesi He, Lihong Wang, Tim Doheny-Adams, Swen Langer, Ian Bancroft

**Affiliations:** Department of Biology, University of York, Heslington, York, YO10 5DD, UK

**Keywords:** Glucosinolates, Genetic associations, Associative transcriptomics, SNP markers, Gene expression markers, Population structure, Oilseed rape, *Brassica napus*

## Abstract

The transcriptome-based GWAS approach, Associative Transcriptomics (AT), which was employed to uncover the genetic basis controlling quantitative variation of glucosinolates in *Brassica napus* vegetative tissues is described. This article includes the phenotypic data of leaf and root glucosinolate (GSL) profiles across a diversity panel of 288 *B. napus* genotypes, as well as information on population structure and levels of GSLs grouped by crop types. Moreover, data on genetic associations of single nucleotide polymorphism (SNP) markers and gene expression markers (GEMs) for the major GSL types are presented in detail, while Manhattan plots and QQ plots for the associations of individual GSLs are also included. Root genetic association are supported by differential expression analysis generated from root RNA-seq. For further interpretation and details, please see the related research article entitled *‘Genetic architecture of glucosinolate variation in Brassica napus’* (Kittipol et al., 2019).

**Table:** Specifications Table

Subject area	Biology
More specific subject area	*Brassica* secondary metabolite and genetics
Type of data	Figure, Tables (MS Excel spreadsheets)
How data was acquired	Glucosinolate measurements were obtained using HPLC on C18 reverse phase column. SNP identification, transcript quantification, construction of the reference coding DNA sequence and associative transcriptomic analysis platform were developed prior to this publication.
Data format	Raw, processed, analyzed
Experimental factors	Desulfoglucosinolates determined as glucosinolates from leaves and roots of genotyped *B. napus* diversity panel. SNP- and GEM-trait association data were analyzed using R scripts.
Experimental features	Transcriptome-based genome wide association
Data source location	Glucosinolate data was collected at the University of York, York, UK.
Data accessibility	Short read sequence data have been deposited at the Sequence Read Archive under BioProject ID: PRJNA524101 (https://www.ncbi.nlm.nih.gov/bioproject/PRJNA524101). Glucosinolate data are provided in Annex spreadsheets.
Related research article	V. Kittipol, Z. He, L. Wang, T. Doheny-Adams, S. Langer, I. Bancroft, Genetic architecture of glucosinolate variation in *Brassica napus*, J. Plant Physiol. 240 (2019) 152988. https://doi.org/10.1016/j.jplph.2019.06.001[1].

**Table:** 

**Value of the data** • This data provides comprehensive leaves and roots glucosinolate profiles across a diversity panel of 288 *Brassica napus* (oilseed rape) genotypes with information on the population structure. Glucosinolate trait data can benefit oilseed rape agribusinesses and researchers of this field in the selection of genotypes with desirable profiles or manipulation of profiles to modulate plant-pest interactions. • The GEM and SNP markers identified in the region of the genome that controls the variation in glucosinolate contents can help accelerate breeding of oilseed rape by marker-assisted selection • This data could be used for comparison or replication of genetic association markers for the natural glucosinolate variations in other populations and other plant tissues.

## 1 Data

**Fig. 1:**
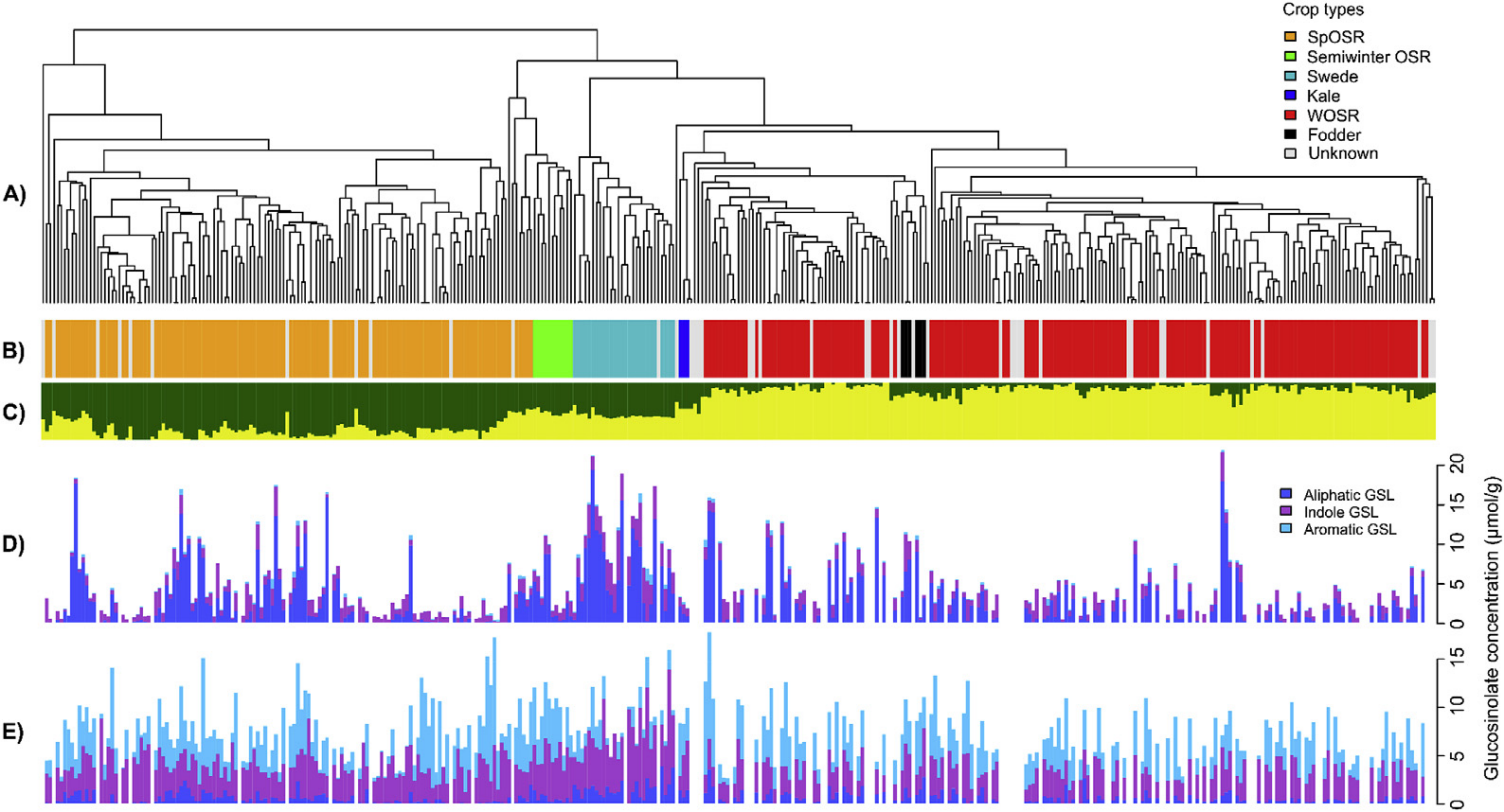
**Population structure and Glucosinolate variation from 288 *B*. *napus* accessions of the Renewable Industrial Products from Rapeseed (RIPR) Panel**. (A) Relatedness of accessions in the panel based on 355 536 scored single-nucleotide polymorphisms (SNPs). (B) Main crop types, color coded: orange for spring oilseed (SpOSR); green for semi-winter oilseed rape; light blue for swede; dark blue for kale; red for winter oilseed rape(WOSR); black for winter fodder and gray for crop type not assigned. (C) Population structure for highest likelihood *k*=2. Variation for glucosinolates content (D) leaf and (E) root of 288 *B*. napus accessions. Individual glucosinolates were grouped according to their structural class as aliphatic (dark blue), indole(margenta) and aromatic(light blue). Panel A, B and C reproduced from Havlickova et al 2018.

The data contains information on leaves and roots glucosinolate (GSL) profiles of 288 *Brassica napus* genotypes (Fig. 1). The relatedness of the accessions was analyzed and visualized by the dendrogram (Fig. 1A). The seven assigned crop types shows the expected clustering (Fig. 1B) with the highest likelihood of two differentiated subpopulations (k = 2), which separated into the spring or winter oilseed rape crop types or a mixture of the two (Fig. 1C). Full dataset of the GSL profiles are presented as mean from four biological replicates of each accessions (Appendix 1) with distribution of the data displayed as histograms (Appendix 2) and analysis of GSL contents by crop types (Appendix 3)*.*

These phenotypic data were used to generate association data identifying single nucleotide polymorphism (SNP) markers and gene expression markers (GEMs) in transcriptome-based genome wide association studies, Associative Transcriptomics (AT) [2], [3]. The Manhattan plots for these associations are shown in Appendix 4 for root traits and Appendix 5 for leaf traits. The significance of the trait associations, shown as –log_10_P value, passing both false discovery rate (FDR) threshold at 5% and threshold for Bonferroni significance of 0.05 suggested that the surrounding genomic region has a strong association with the trait. To assess how well the model accounts for population structure and familial relatedness, quantile-quantile (QQ) plots from SNP association analyses have been generated (Appendix 6 & Appendix 7). Appendix 8 summarizes the optimal algorithm showing calculated group kinship matrix, 2*log likelihood function and the estimated heritability for all GSL traits.

As shown in Fig. 1, aliphatic GSLs is the most abundant class of GSL in *B. napus* leaves. SNP-based associations of leaf aliphatic GSL revealed strong associations with markers in the defined regions of chromosome A2, A9, C2, C7 and C9 (Appendix 9). Within these data tables, details of trait associations for genome-assigned markers are provided, including polymorphism, significance of association and the frequency of the minor allele in the population. The same associated regions were shown for total seed GSL in *B. napus* (Appendix 10). As presented in [1], orthologues of *HAG1* (AT5G61420), a transcription factor that positively regulated aliphatic GSL biosynthesis, have been discovered within all of these SNP-based associated loci (Appendix 9). In addition, the six GEMs detected above the threshold for the false discovery rate (FDR) at 5% are shown to be involved directly in aliphatic GSL biosynthesis, with orthologues of *HAG1* as the top GEMs (Appendix 11). Presence of GEM association peaks on chromosome A9, C2 and C9 for aliphatic GSL suggested structural genome variation via homoeologous exchange where neighboring genes displayed the same directionality of one genome over-expressed and other genome under-expressed (Appendix 12). The Transcriptome Display Tile Plots [4] was used to visualize the homoeologous exchanges in these regions (Appendix 13).

In *B. napus* roots, aromatic GSL is the dominant GSL class and revealed a clear SNP association peak on chromosome A3 (Appendix 4). As described in [1], an orthologue of *HAG3* was identified in close proximity to the top associated SNP markers within in this region (Appendix 14). To support gene expression analysis in roots, differential expression analysis from root transcriptome-sequence was performed, which compared the expression patterns of 4 accessions with high root aromatic GSLs and 4 accessions with low root aromatic GSLs (Appendix 15). Within the SNP associated region of chromosome A3, *Bna.HAG3.A3* showed the highest significant log_2_ fold-change (Appendix 15) with higher expression of *Bna.HAG3.A3* observed in high-root aromatic GSL genotypes and vice versa in the low-root GSL genotypes. To limit potential confounding effect between GSL pathways, further stringent analysis of differential root expression (p ≤ 1 × 10^−10^) was performed between accessions which differs in root aromatic GSLs but are low in aliphatic GSLs (Appendix 16). This analysis revealed insight into genes that had been identified in aliphatic GSL pathway but could have potential roles in the aromatic GSL pathway. This is shown by the significant positive correlations between their expression levels and levels of aromatic GSL (Appendix 17).

To investigate the relationship of GSLs between vegetative tissues and seeds, seed GSL data from [5] was added to the dataset. Correlation analysis between levels of aliphatic GSLs and the transcript abundance of GSL transporters, *GTR1* (AT3G47960) and *GTR2* (AT5G62680), was conducted to investigate the role of transporters on GSL accumulation pattern (Appendix 18), as described in [1]. Finally, correlations between leaf and seed GSLs was analyzed to investigate the basis for the accumulation pattern of GSLs in these tissues (Appendix 19).

## 2 Experimental design, materials, and methods

### 2.1 Growth of plant material for glucosinolate content

A subset of 288 *B. napus* accessions from the Renewable Industrial Products from Rapeseed (RIPR) diversity population [2] was grown in long day (16/8 h, 20 °C/14 °C) under controlled glasshouse conditions (University of York, UK). Within this panel, there are 56 Modern Winter OSR, 65 Winter OSR, 6 Winter Fodder, 121 Spring OSR, 26 Swede and 14 Exotic varieties (Appendix 1). Four biological replicates of each accession were grown in root trainers with Terra-Green for ease of root harvesting, supplemented weekly with a half concentration of Murashige and Skoog growth medium [6] adjusted to pH6.5 with KOH. The experiment was arranged as randomized four-block design with one plant per lines in each block. Four weeks after sowing, the third true leaf and the whole root system were harvested from each plant. At harvest, leaves were cut at the base, wrapped in a labelled aluminum foil and immediately frozen in liquid nitrogen. Plants were removed from the tray, had the roots washed, dried with paper towel and cut. All samples were wrapped in labelled aluminum foils and immediately frozen in liquid nitrogen and stored at −80 °C.

### 2.2 Glucosinolate quantification

As per the recommended quantification method previously tested [7], frozen tissue samples were lyophilized before homogenized to fine powder for 10 min at a frequency of 30 Hz (TissueLyser II, Qiagen). To 50 mg of homogenate, 1975 μl of 80% (v/v) methanol and 25 μl of 5 mM internal standard glucotropaeolinwas added. The sample was mixed and left to stand for 30 min at 20 °C and further mixed with orbital shaker (Vibrax, IKA) at 1200 rpm for 30 min before centrifugation at 8000 rpm for 10 min. Supernatant methanol extract was then transferred to the pre-conditioned Sephadex column in purification step. Purification and desulfation of GSLs was according to [8]. Columns were prepared with 0.5 ml ion-exchange resin (DEAE Sephadex beads in 1:1 ratio with 2 M acetic acid), conditioned with 2 ml imizadoleformate (6 M) and washed twice with 1 ml water. One ml of the extract was transferred to a prepared column and gently washed twice with 1 ml 20 mM sodium acetate (pH 4) before adding 75 μl of purified sulfatase (5 U/ml). Columns were incubated for 24 h and desulfoglucosinolates were eluted with two 1 ml portions of water.

Desulfoglucosinolates were separated by HPLC (Millipore 600E system, Waters) on a reverse phase C18 column at 30 °C (Phenomonex, SphereClone 5μ ODS(2), 150 mm × 4.6 mm) with mobile phase solutions consisting of 100% diH_2_O and 30% (v/v) acetronitile, as detailed in [7]. Injection was at 10 μl and flow rate was set to 1 ml/min. The absorbance of the eluates was monitored at 229 nm wavelength within the UV spectrum. Samples were separated according to the program described in [7]. Through standard injections, HPLC-MS identification, retention time and photodiode array (PDA) UV spectra, the identity of all major GSLs were confirmed.

### 2.3 Statistical analysis

Statistical analyses were carried out with R statistical software [9]. One-way ANOVA and Tukey's honest significant difference (HSD) post hoc test were performed on GSL content between crop types (Appendix 3).

### 2.4 Transcriptome sequencing, SNP identification and transcript quantification

Plant growth conditions, sampling of material, RNA extraction and Illumina transcriptome sequencing was carried out and described previously in [4]. For each genotype, RNA-sequence data was mapped onto recently developed ordered Brassica A and C genome-based pan-transcriptomes as reference sequences [10], using the methods described in [11]. SNP positions were excluded from the alignment if they have a read depth below 10, a base call quality below Q20, missing data below 0.25, and three alleles or more. After rigorous filtering and quality checking parameters to reduce errors in SNP identification and assessment of linkage disequilibrium as detailed in [2], a set of 355 536 SNP markers was generated, of which 256 397 SNP had a minor allele frequency (MAF) > 0.01. Transcript abundance was quantified and normalized as reads per kb per million aligned reads (RPKM) for each sample. Of the 116 098 coding DNA sequence (CDS) models, 53 889 CDS models was detected with significant expression (>0.4 RPKM).

### 2.5 Associative Transcriptomics

**Fig. 2:**
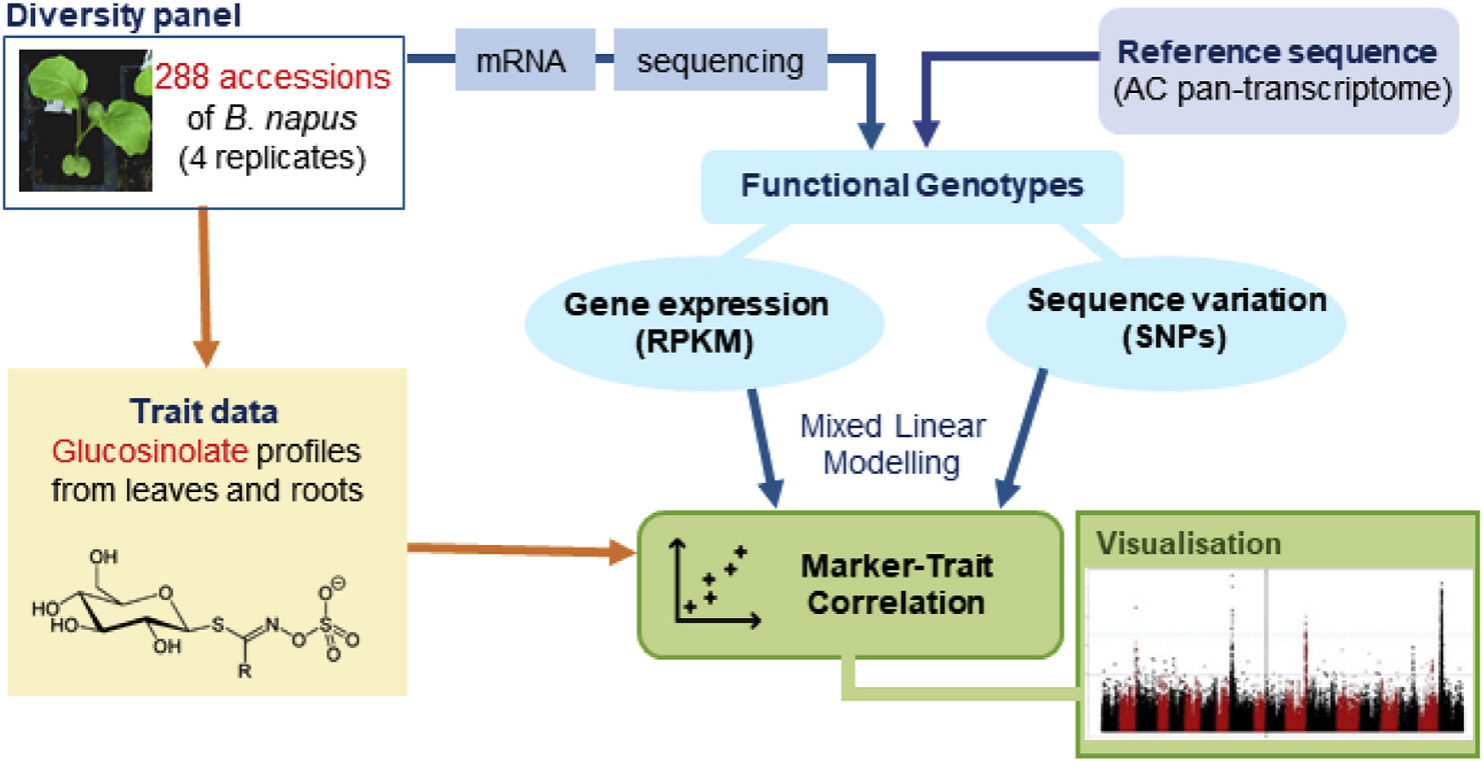
Overview of associative transcriptomic analysis.

An overview of Associative Transcriptomics (AT) analysis is shown in Fig. 2. The use of transcriptome sequencing in AT allows the discovery of SNP markers in tight linkage disequilibrium with causative genes like conventional GWAS, with the additional feature of finding genes with expression patterns (gene expression markers, GEM) that correlate with the trait variation.

AT was performed using R [9] based on an adaption of the first AT methods [3] with modifications to accommodate for larger dataset, as detailed in [2]. To reduce the risks of false positive associations from undetected population structure that can mimic the signal of association, population structure inference using kernel-PCA and optimization (PSIKO; highest likelihood subpopulation k = 2) [12] was used for Q-matrix generation to correct for population stratification. SNP-based analyses were performed with Genome Association and Prediction Integrated Tool (GAPIT) R package using mixed linear model that includes both fixed and random effects [13]. SNP markers with minor allele frequencies below 0.01 were removed from the SNP dataset leaving 256 397 SNPs for the associations [2]. SNP markers that can be assigned with confidence to the genomic position of the CDS model are rendered dark points and markers that could not be assigned with confidence were rendered pale points. For GEM-based analyses, fixed-effect linear model was calculated in R software, with RPKM values and the Q matrix inferred by PSIKO as explanatory variables, and trait score as the response variable [2]. For each regression, coefficients of determination (R^2^), constant, F-value and significance P-values were produced. When genomic inflation factor (λ) was >1, genomic control with P-value adjustment [14] was applied to the GEM analysis to correct for false associations. False discovery rate (FDR) [15] and threshold for Bonferroni [16] corrections were used to set significance threshold at P < 0.05. Quantile-Quantile plots all association analyses are included as Appendix 6 for root data and Appendix 7 for leaf data.

### 2.6 Differential expression analysis of root RNA-seq data

Differential gene expression was analyzed using root transcriptome sequences from four biological replicates (i.e. using root RNA-seq from 4 separate plants of each plant type). The methods in Bioconductor package EdgeR [17] were used to identify the differential expressed genes. In multiple comparisons, both fold change (FC) > 2 and false discovery rate (FDR) < 0.05 were used to flag a gene being differentially expressed. Flags of “1”,“-1” and “0” were used to note positively, and negatively or not significantly expressed genes in the data and can be filtered among comparisons.

## References

[1] Kittipol V., He Z., Wang L., Doheny-Adams T., Langer S., Bancroft I. (2019). Genetic architecture of glucosinolate variation in Brassica napus. J. Plant Physiol..

[2] Havlickova L., He Z., Wang L., Langer S., Harper A.L., Kaur H., Broadley M.R., Gegas V., Bancroft I. (2018). Validation of an updated Associative Transcriptomics platform for the polyploid crop species Brassica napus by dissection of the genetic architecture of erucic acid and tocopherol isoform variation in seeds. Plant J..

[3] Harper A.L., Trick M., Higgins J., Fraser F., Clissold L., Wells R., Hattori C., Werner P., Bancroft I. (2012). Associative transcriptomics of traits in the polyploid crop species Brassica napus. Nat. Biotechnol..

[4] He Z., Wang L., Harper A.L., Havlickova L., Pradhan A.K., Parkin I.A.P., Bancroft I. (2016). Extensive homoeologous genome exchanges in allopolyploid crops revealed by mRNAseq-based visualization. Plant Biotechnol. J..

[5] Lu G., Harper A.L., Trick M., Morgan C., Fraser F., O'Neill C., Bancroft I. (2014). Associative transcriptomics study dissects the genetic architecture of seed glucosinolate content in Brassica napus. DNA Res..

[6] Murashige T., Skoog F. (1962). A revised medium for rapid growth and bio assays with tobacco tissue cultures. Physiol. Plant..

[7] Doheny-Adams T., Redeker K., Kittipol V., Bancroft I., Hartley S.E. (2017). Development of an efficient glucosinolate extraction method. Plant Methods.

[8] ISO 9167-1 (1992). Determination of Glucosinolates Content - Part 1: Method Using High-Performance Liquid Chromatography.

[9] R core team (2013). R: a Language and Environment for Statistical Computing.

[10] He Z., Cheng F., Li Y., Wang X., Parkin I.A.P., Chalhoub B., Liu S., Bancroft I. (2015). Construction of Brassica A and C genome-based ordered pan-transcriptomes for use in rapeseed genomic research. Data Br.

[11] Bancroft I., Morgan C., Fraser F., Higgins J., Wells R., Clissold L., Baker D., Long Y., Meng J., Wang X., Liu S., Trick M. (2011). Dissecting the genome of the polyploid crop oilseed rape by transcriptome sequencing. Nat. Biotechnol..

[12] Popescu A.A., Harper A.L., Trick M., Bancroft I., Huber K.T. (2014). A novel and fast approach for population structure inference using Kernel-PCA and optimization. Genetics.

[13] Lipka A.E., Tian F., Wang Q., Peiffer J., Li M., Bradbury P.J., Gore M.A., Buckler E.S., Zhang Z. (2012). GAPIT: genome association and prediction integrated tool. Bioinformatics.

[14] Devlin B., Roeder K. (1999). Genomic control for association studies. Biometrics.

[15] Benjamini Y., Hochberg Y. (1995). Controlling the false discovery rate: a practical and powerful approach to multiple testing. J. R. Stat. Soc..

[16] Dunn O.J. (1961). Multiple comparisons among means. J. Am. Stat. Assoc..

[17] Robinson M.D., McCarthy D.J., Smyth G.K. (2009). edgeR: A Bioconductor package for differential expression analysis of digital gene expression data. Bioinformatics.

