# Supplementary material for: Data in support of genetic architecture of glucosinolate variations in Brassica napus
Source: Data Brief. 2019 Aug 14;25:104402. doi: 10.1016/j.dib.2019.104402 (PMC6722234; doi:10.1016/j.dib.2019.104402)
Supplement: Supplementary file 1 [file mmc1.zip › Appendix12_rpkmGEM_A9C9.pdf]

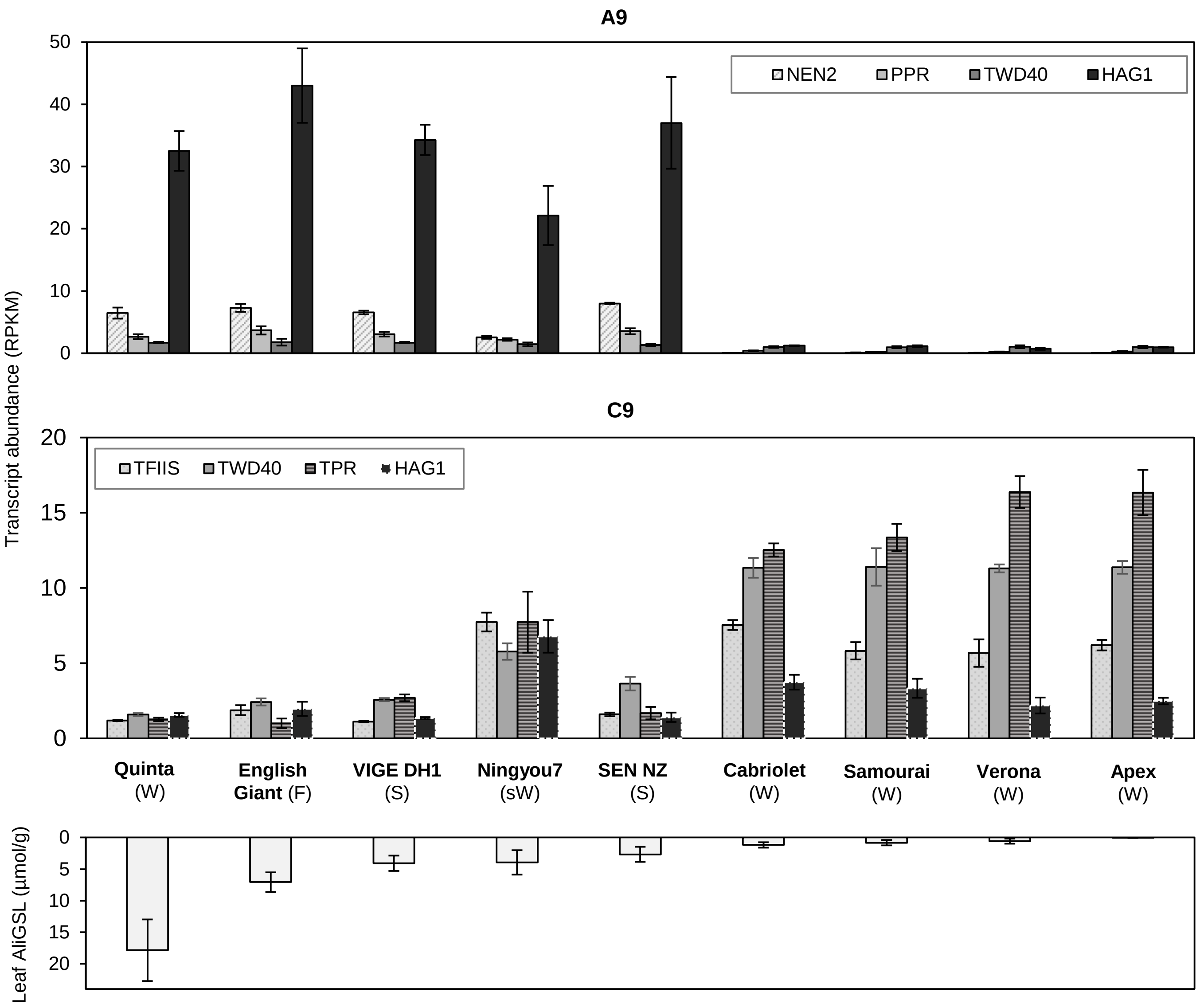

Appendix 12. Comparison of the top GEM markers transcript abundance on chromosome A9 and C9 between high- and low- leaf aliphatic glucosinolate *B. napus* cultivars. Transcript abundance is expressed as reads per kb per million aligned reads (RPKM), with error bars to indicate standard deviation from four biological replicates of each accessions. Orthologue of *HAG1* on chromosome C9 is not one of the top markers but included for comparison. Crop type abbreviation: W, Winter oilseed rape; F, Winter fodder; S, Swede; sW, Semiwinter oilseed rape.
