# Supplementary material for: Data in support of genetic architecture of glucosinolate variations in Brassica napus
Source: Data Brief. 2019 Aug 14;25:104402. doi: 10.1016/j.dib.2019.104402 (PMC6722234; doi:10.1016/j.dib.2019.104402)
Supplement: Supplementary file 1 [file mmc1.zip › Appendix13_TilePlot.pdf]

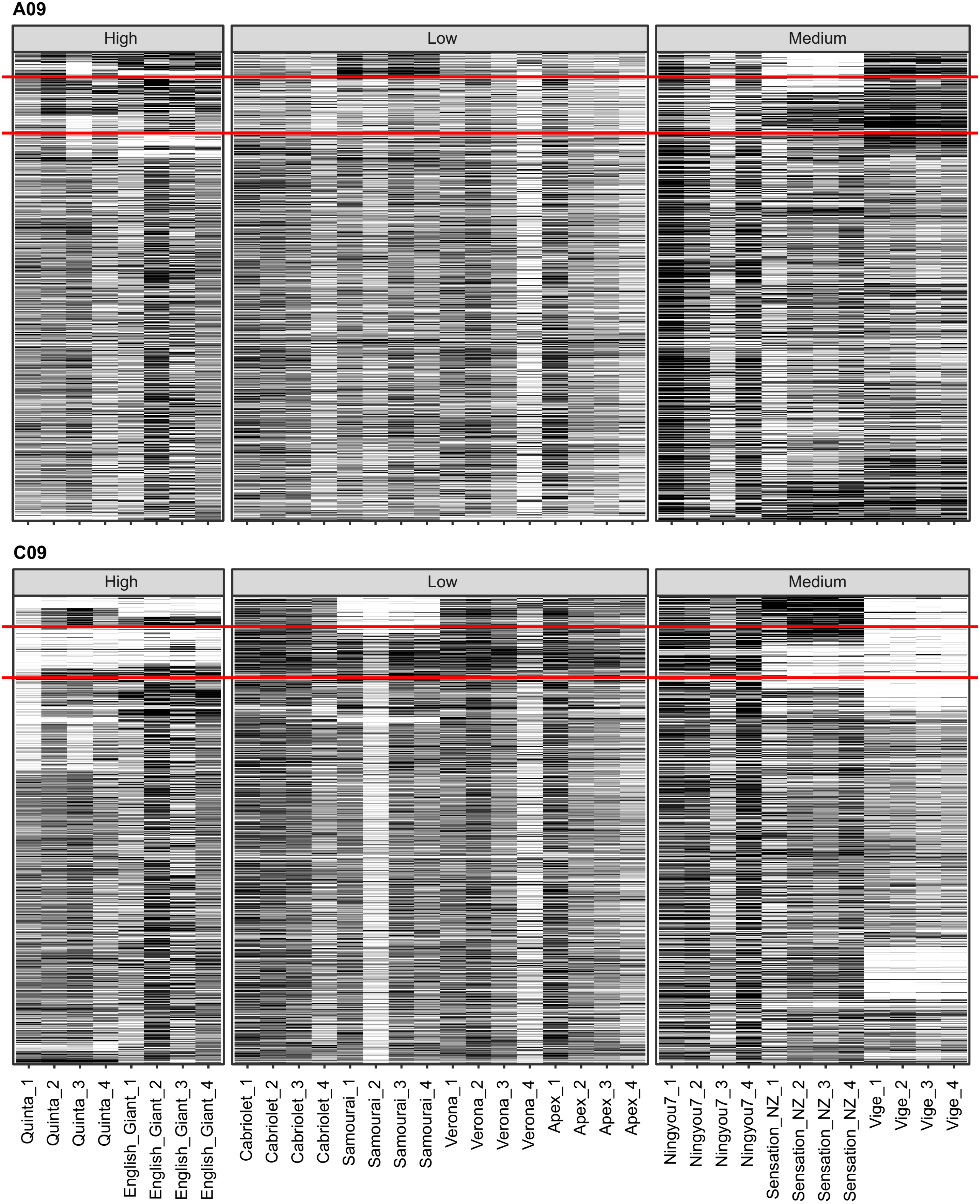

Appendix 13. Transcriptome Display Tile Plots illustrates homoeologous genome exchange between A9 and C9 in *Brassica napus*, based on mRNA sequence data. The top plot shows the relative transcript abundance of A genome on chromosome A9, the bottom plot shows the relative transcript abundance of C genome on chromosome C9, plotted with four biological cultivar replicates. Darker or lighter shade of grey represent increased or decreased gene expression. Inverted colour of the same region on the different chromosome show homoeologous exchange. The positions of GEM association peaks on the top of A9 and C9 are marked with two horizontal red lines. Cultivars are grouped according to the total aliphatic GSL content in the leaf : high (>7 µmol/g), medium (2 - 7 µmol/g) and low (<2 µmol/g).
