# Supplementary material for: Data in support of genetic architecture of glucosinolate variations in Brassica napus
Source: Data Brief. 2019 Aug 14;25:104402. doi: 10.1016/j.dib.2019.104402 (PMC6722234; doi:10.1016/j.dib.2019.104402)
Supplement: Supplementary file 1 [file mmc1.zip › Appendix17_RPKMcorr_RootAro.pdf]

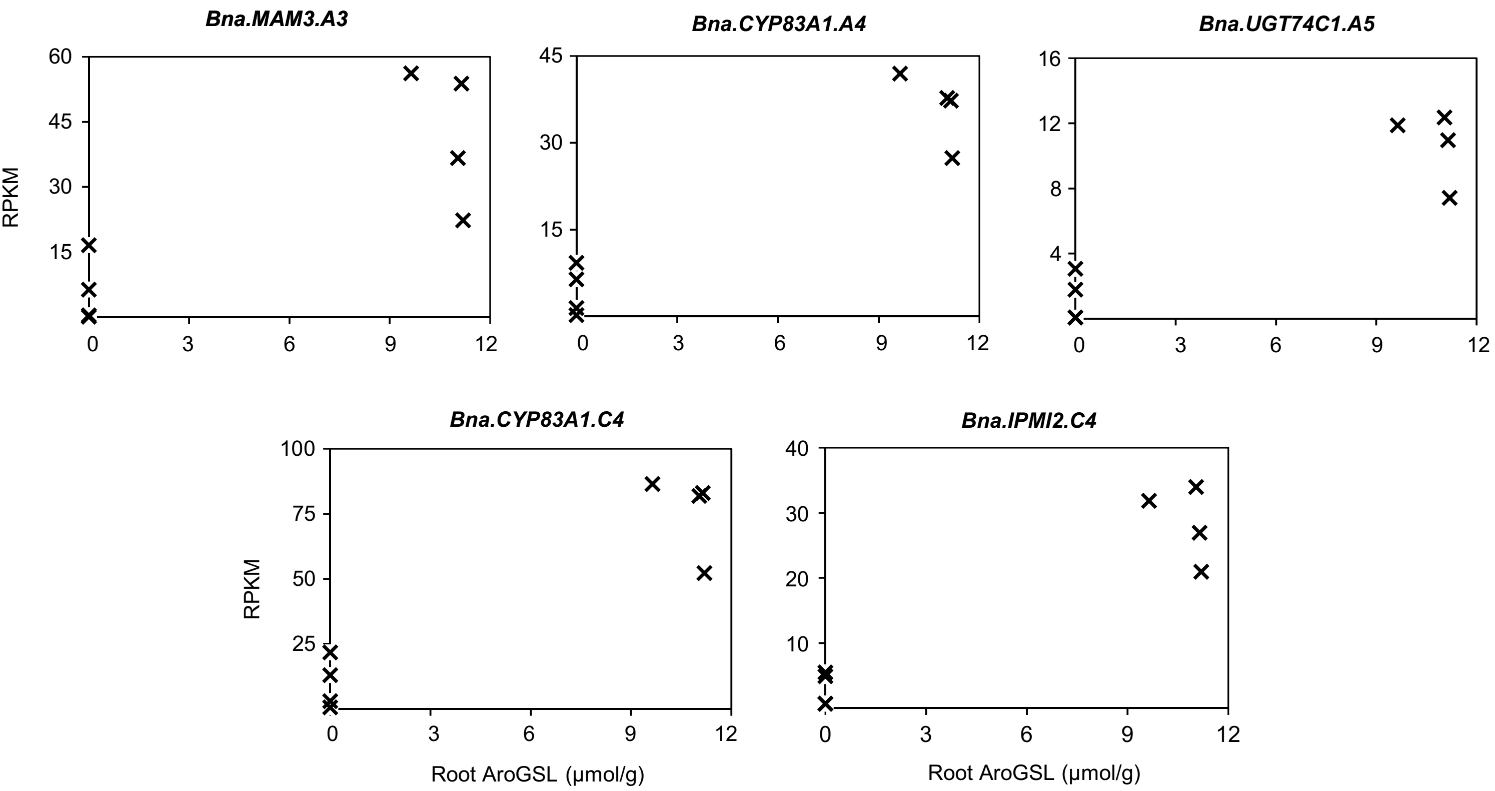

Appendix 17. Correlation of root transcript abundance with root aromatic glucosinolate. These genes, identified from the root differential expression analysis, are known to be involved in the glucosinolate biosynthetic pathway. Average of four biological replicates were shown. *Orthologues of MAM3* (AT5G23020), *CYP83A1* (AT4G13770), *UGT74C1* (AT2G31790), *IPMI2* (AT2G43100).
