# Supplementary material for: Data in support of genetic architecture of glucosinolate variations in Brassica napus
Source: Data Brief. 2019 Aug 14;25:104402. doi: 10.1016/j.dib.2019.104402 (PMC6722234; doi:10.1016/j.dib.2019.104402)
Supplement: Supplementary file 1 [file mmc1.zip › Appendix18_GTR.pdf]

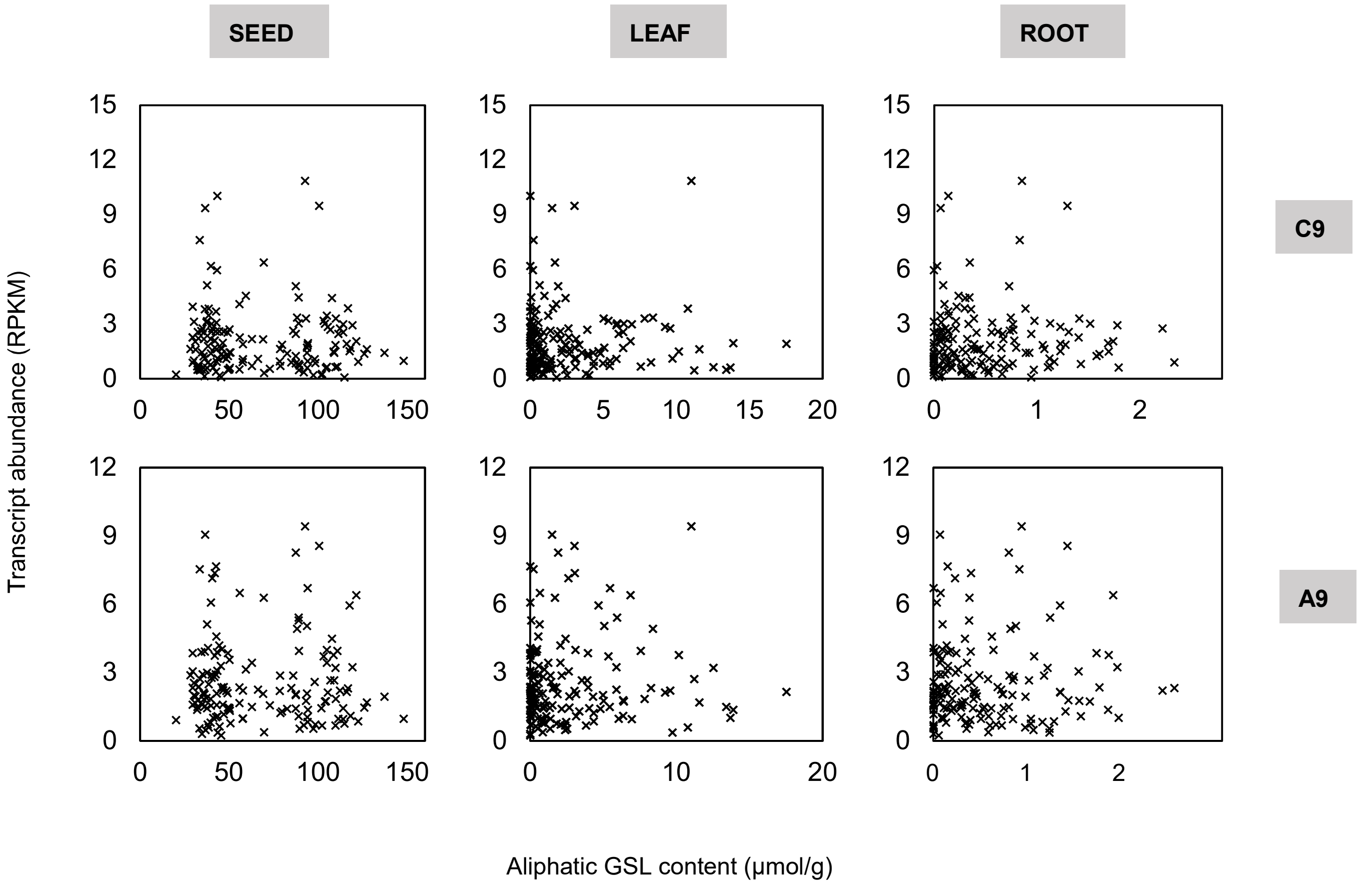

Appendix 18. Correlation of glucosinolate transporters, *Bna.GTR2.A9* and *Bna.GTR2.C9*, transcript abundance with levels of aliphatic glucosinolates in leaves, roots and seeds. Transcript abundance was quantified and normalised as reads per kb per million aligned reads (RPKM). No correlation between gene expressions and changes in levels of aliphatic GSL was observed across all three tissues.
