# Supplementary material for: Data in support of genetic architecture of glucosinolate variations in Brassica napus
Source: Data Brief. 2019 Aug 14;25:104402. doi: 10.1016/j.dib.2019.104402 (PMC6722234; doi:10.1016/j.dib.2019.104402)
Supplement: Supplementary file 1 [file mmc1.zip › Appendix2_Histograms.pdf]

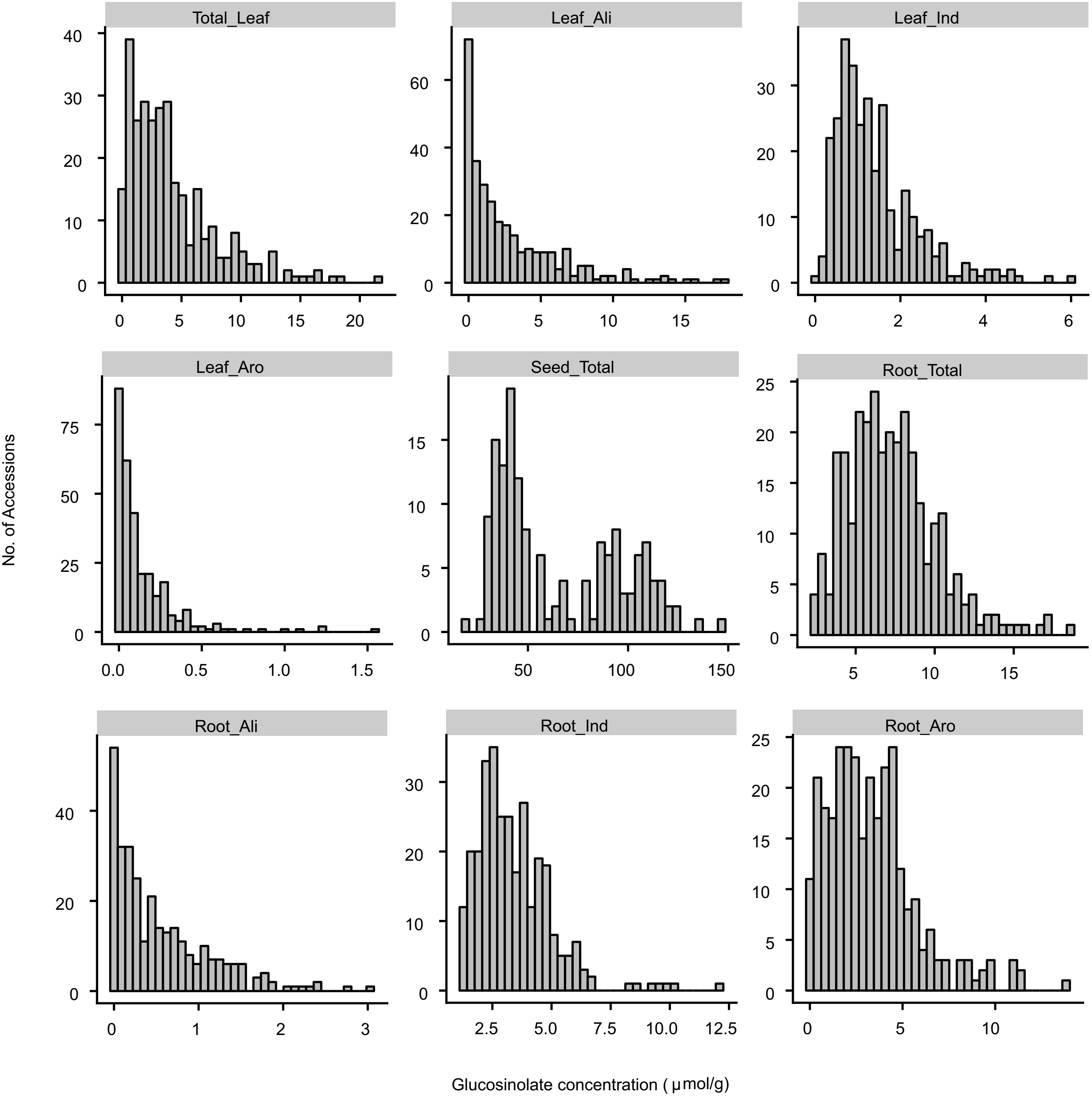

Appendix 2. Histograms of the distribution of glucosinolate structural classes in leaf and root of 288 *B. napus* and total seed glucosinolates distribution of 101 accession from Lu *et al* (2014).

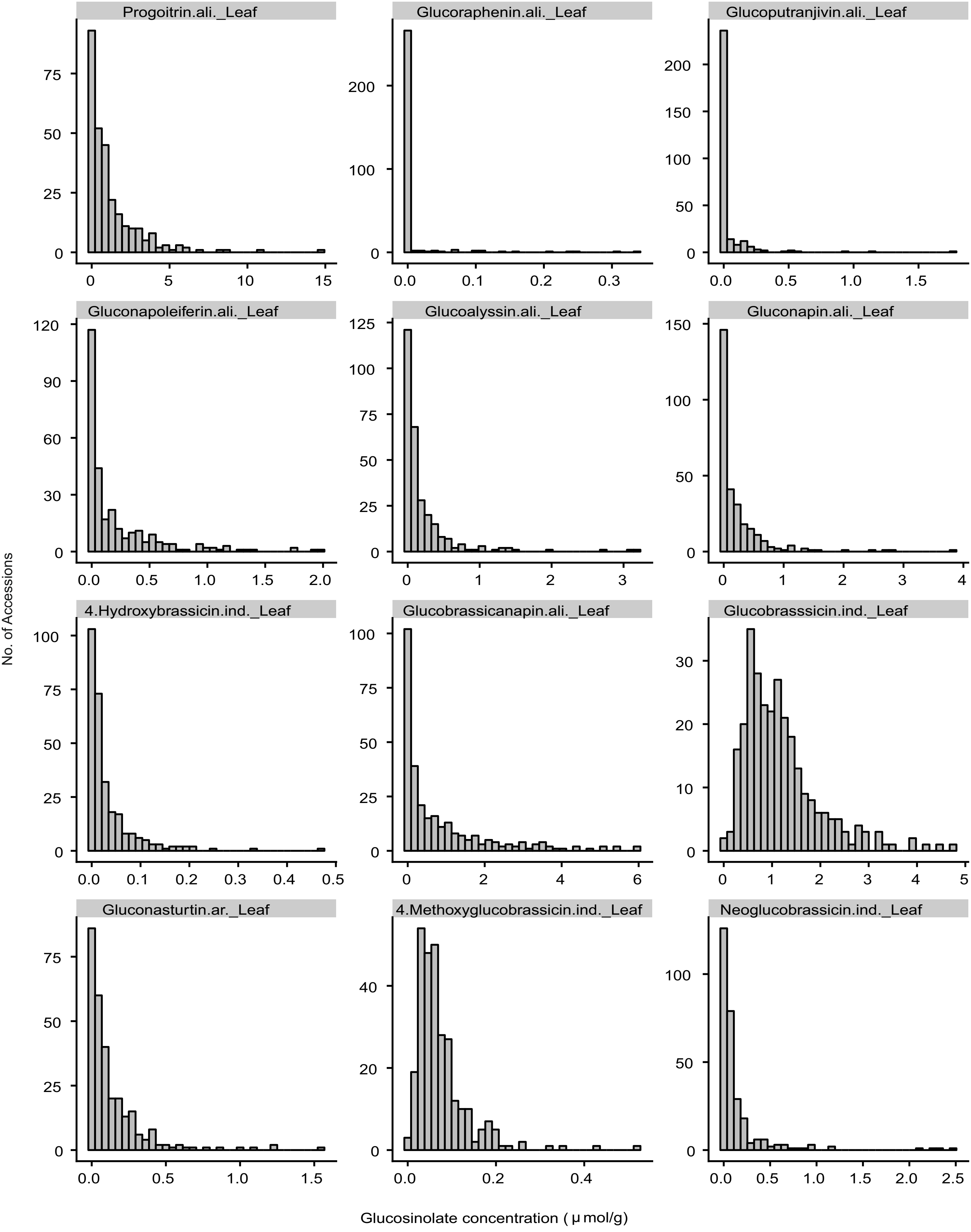

Appendix 2. Histograms of twelve individual glucosinolates composition found in leaf of 288 *B. napus* accessions

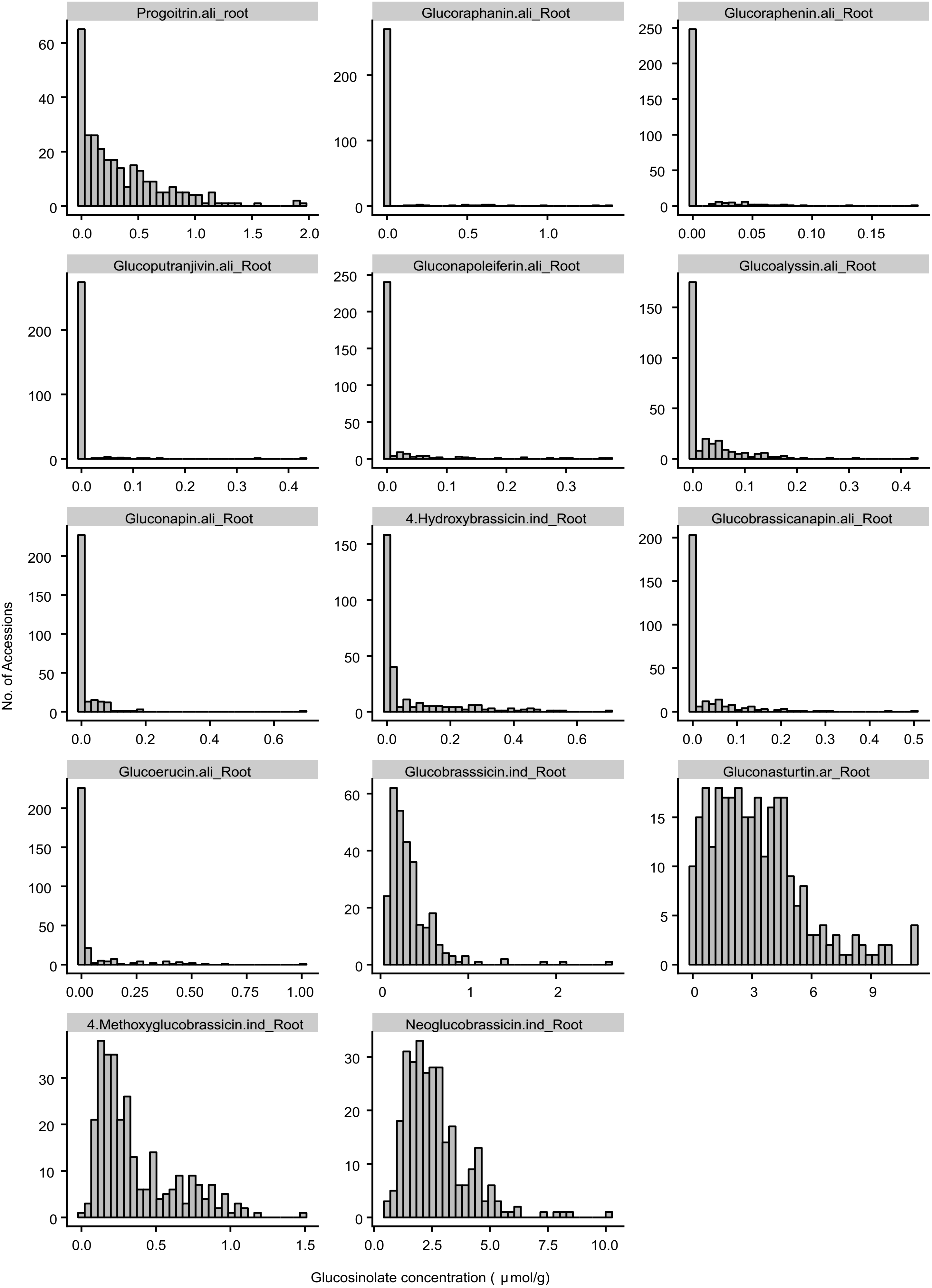

Appendix 2. Histograms of individual glucosinolates compositions in root of 288 *B. napus* accession.
