# Supplementary material for: Data in support of genetic architecture of glucosinolate variations in Brassica napus
Source: Data Brief. 2019 Aug 14;25:104402. doi: 10.1016/j.dib.2019.104402 (PMC6722234; doi:10.1016/j.dib.2019.104402)
Supplement: Supplementary file 1 [file mmc1.zip › Appendix3_Boxplot_croptype.pdf]

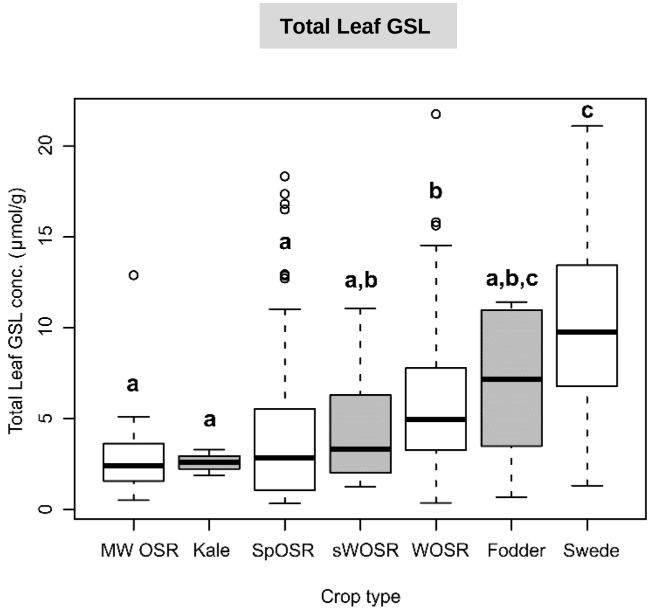

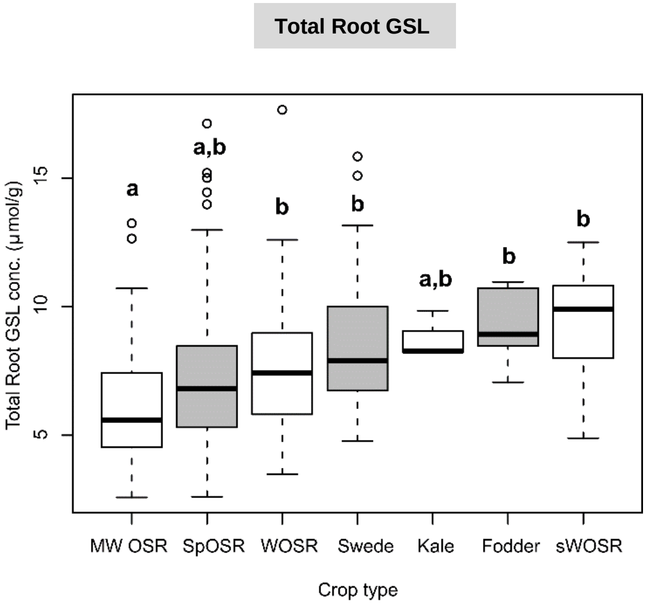

Appendix 3. Total glucosinolate content differs between crop types of 288 *B. napus* accessions. Box-whisker plots of total glucosinolate content in the leaves and roots. The box represents the upper and lower quartiles and the dark line is the median value. The whisker extend to the maximum and minimum values. Open circles indicate outliers. Same letter indicate no significant difference between the groups (Dunnett T3 as post-hoc test for unequal variance and sample size between crop types, $p > 0.05$). Abbreviation: MW OSR, Modern winter oilseed rape; SpOSR, Spring oilseed rape; WOSR, Winter oilseed rape; sWOSR, semiwinter oilseed rape, Fodder, Winter fodder.

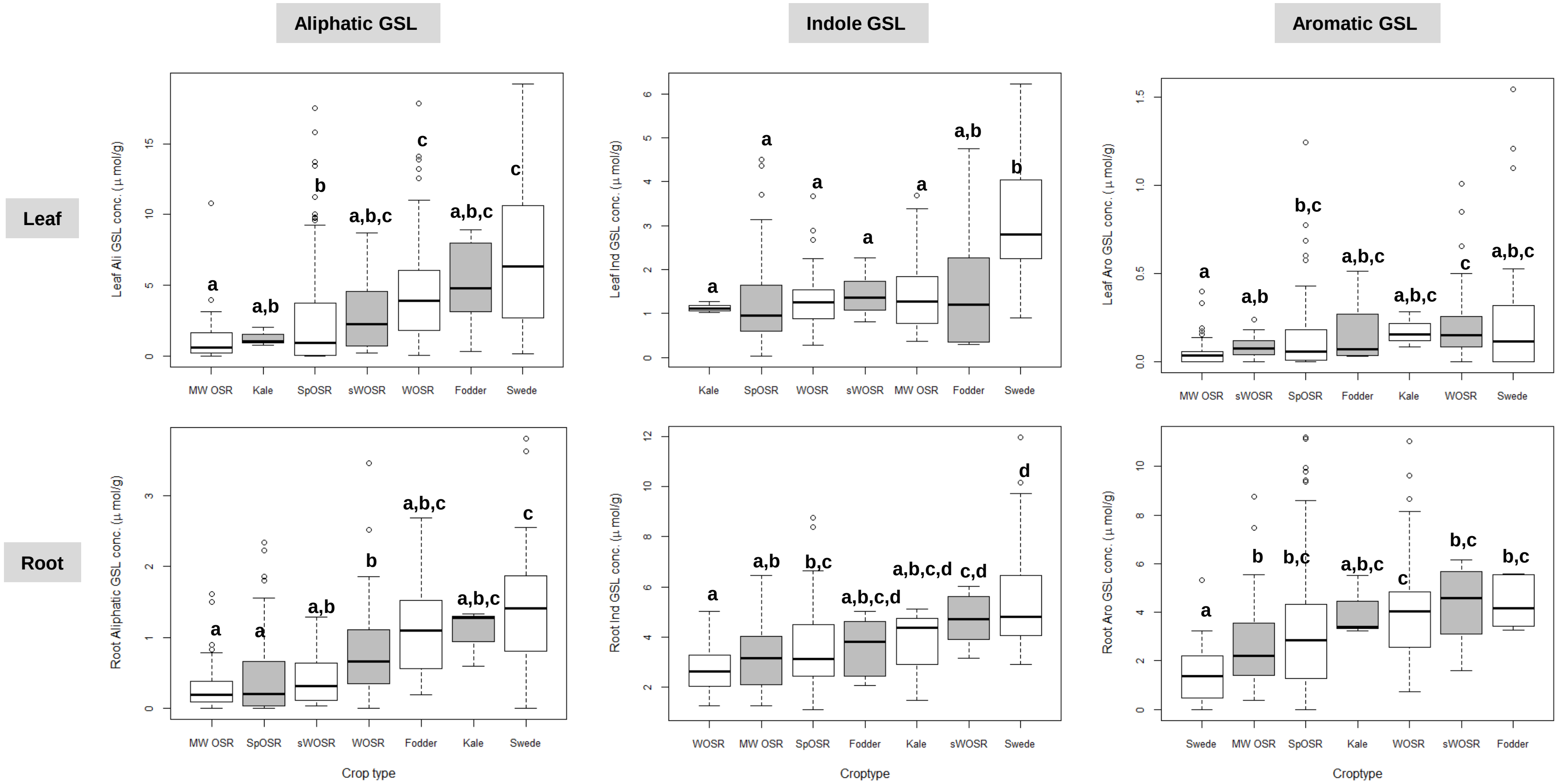

Appendix 3. Comparison of glucosinolate contents between different crop types of 288 *B. napus* accessions. Box-whisker plots of the three structural group of glucosinolates in leaves and roots. The box represents the upper and lower quartiles and the dark line is the median value. The whisker extend to the maximum and minimum values. Open circles indicate outliers. Same letter indicate no significant difference between the groups (ANOVA with Dunnett T3 as post-hoc test for unequal variance and sample size between crop types, p >0.05). Abbreviation: MW OSR, Modern winter oilseed rape; SpOSR, Spring oilseed rape; WOSR, Winter oilseed rape; sWOSR, semiwinter oilseed rape, Fodder, Winter fodder.
