# Supplementary material for: Data in support of genetic architecture of glucosinolate variations in Brassica napus
Source: Data Brief. 2019 Aug 14;25:104402. doi: 10.1016/j.dib.2019.104402 (PMC6722234; doi:10.1016/j.dib.2019.104402)
Supplement: Supplementary file 1 [file mmc1.zip › Appendix5_AllLeafAT.pdf]

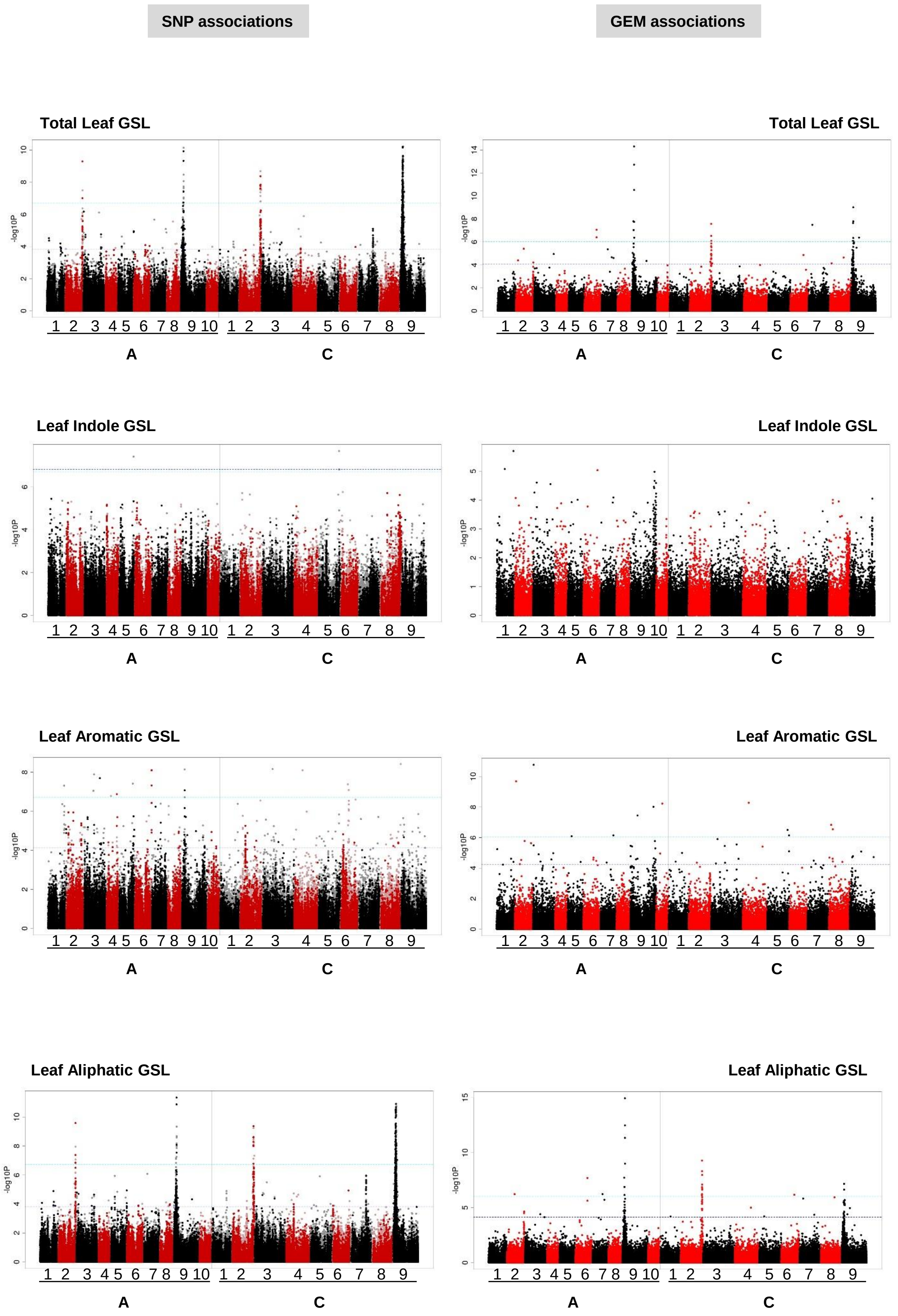
SNP associations
GEM associations
Total Leaf GSL
Total Leaf GSL
Leaf Indole GSL
Leaf Indole GSL
Leaf Aromatic GSL
Leaf Aromatic GSL
Leaf Aliphatic GSL
Leaf Aliphatic GSL
-log10P
1 2 3 4 5 6 7 8 9 10 1 2 3 4 5 6 7 8 9
A
C

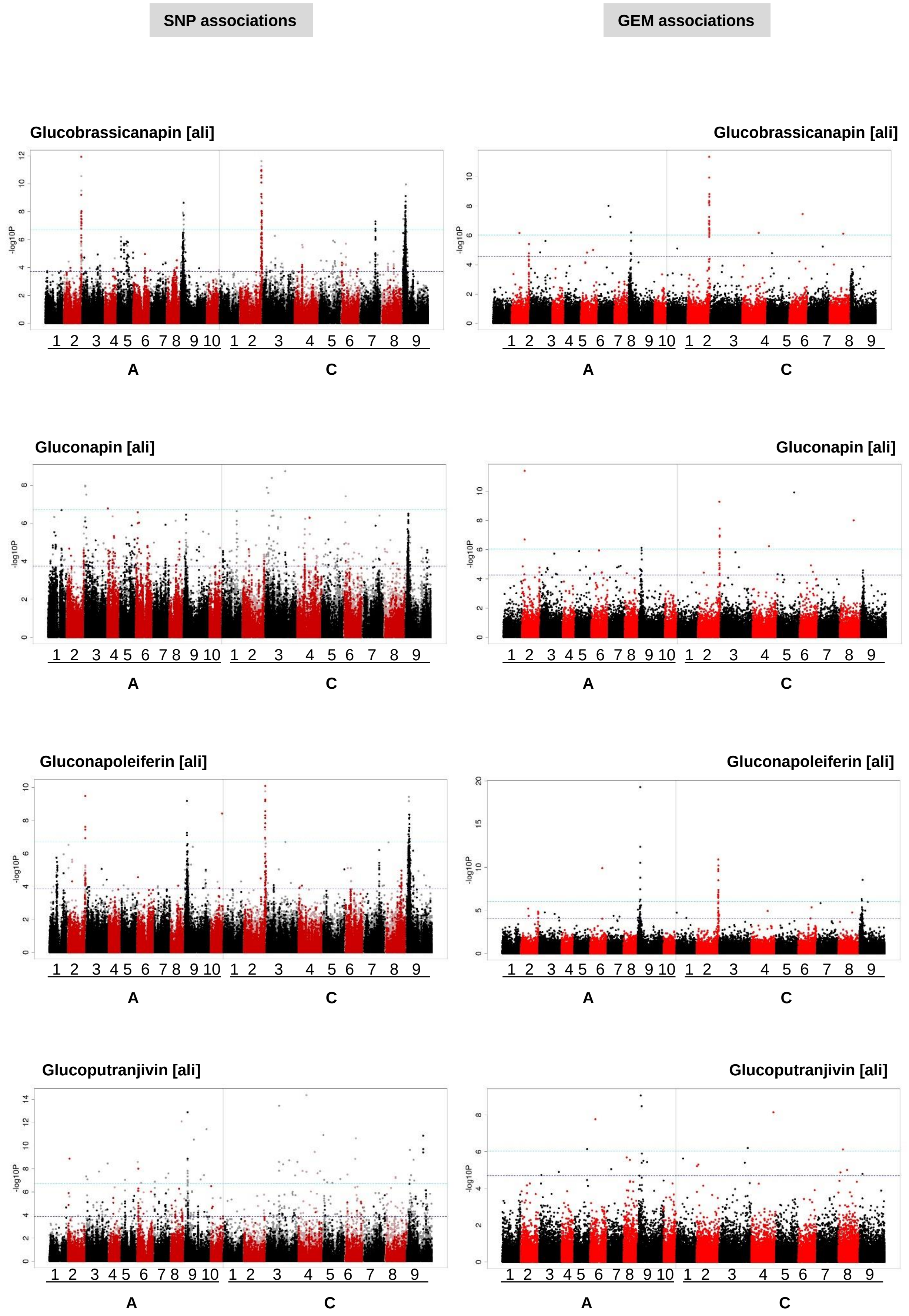
SNP associations
GEM associations
Glucobrassicanapin [ali]
Glucobrassicanapin [ali]
Gluconapin [ali]
Gluconapin [ali]
Gluconapoleiferin [ali]
Gluconapoleiferin [ali]
Glucoputranjivin [ali]
Glucoputranjivin [ali]
-log10P
1 2 3 4 5 6 7 8 9 10 1 2 3 4 5 6 7 8 9
A
C

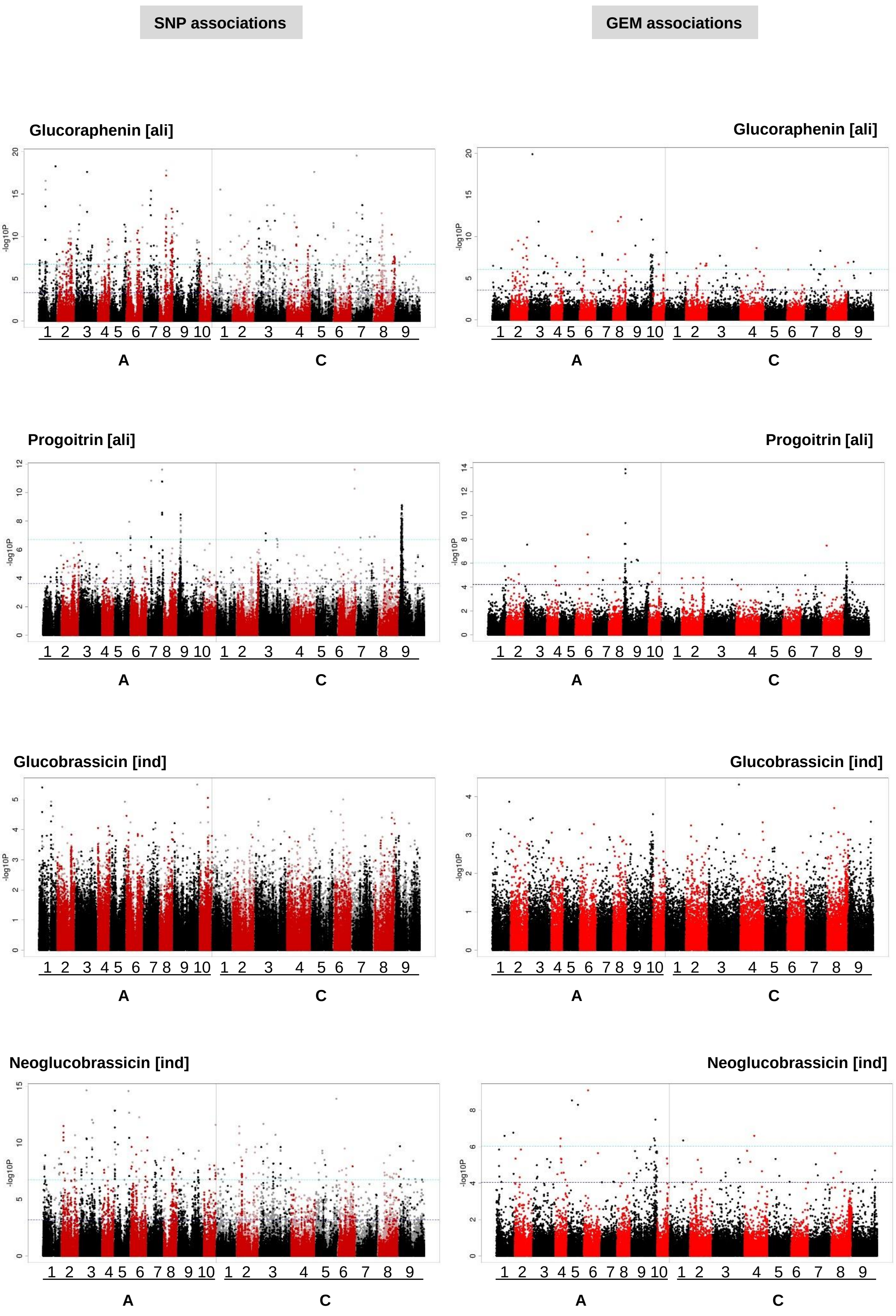
SNP associations
GEM associations
Glucoraphenin [ali]
Glucoraphenin [ali]
Progoitrin [ali]
Progoitrin [ali]
Glucobrassicin [ind]
Glucobrassicin [ind]
Neoglucobrassicin [ind]
Neoglucobrassicin [ind]
-log10P
1 2 3 4 5 6 7 8 9 10 1 2 3 4 5 6 7 8 9
A
C

SNP associations

GEM associations

4-Hydroxybrassicin [ind]

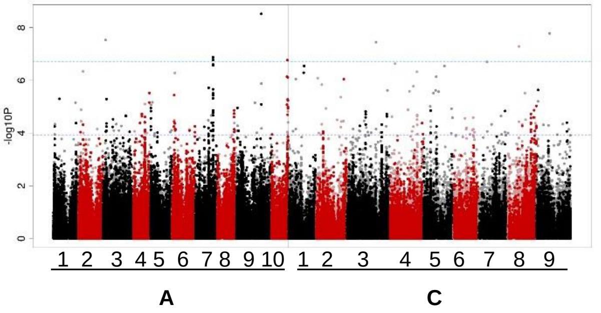

4-Hydroxybrassicin [ind]

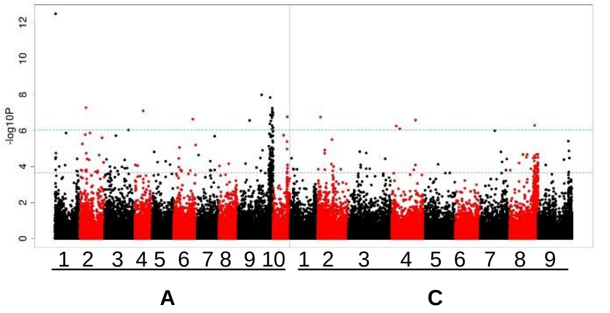

4-Methoxyglucobrassicin [ind]

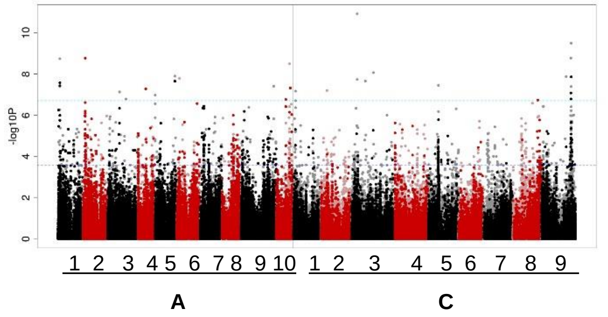

4-Methoxyglucobrassicin [ind]

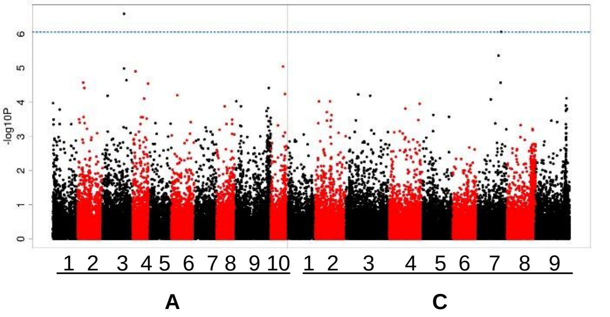

Gluconasturtin [aro]

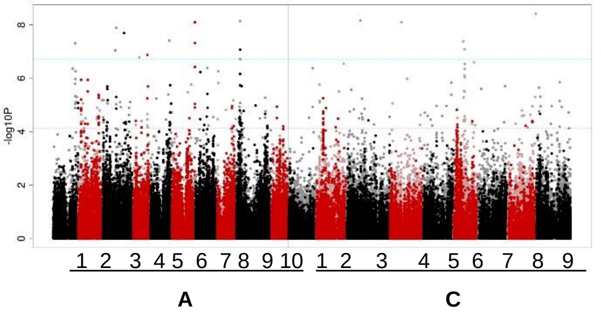

Gluconasturtin [aro]

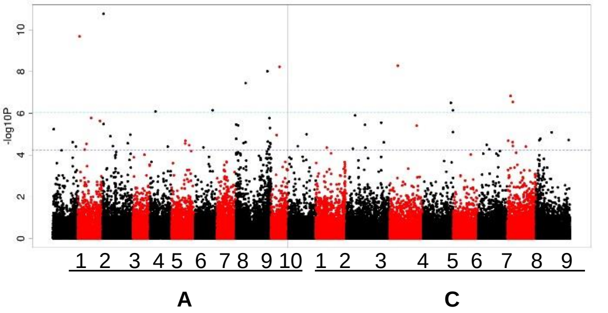

Glucoalyssin [ali]

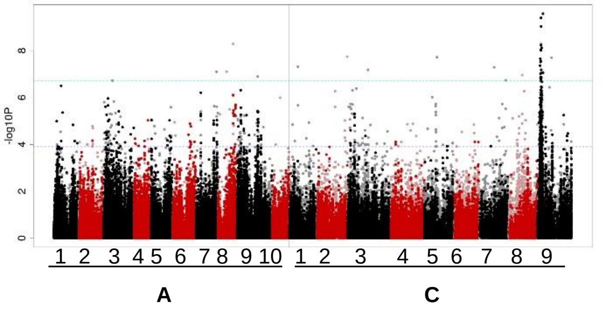

Glucoalyssin [ali]

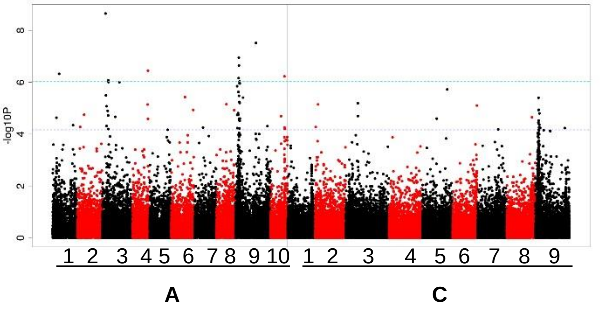

Appendix 5. Association analysis of all glucosinolates (GSL) found in the leaf. Absolute amount of GSL (µmol/g) were used as traits for 288 accessions. The SNP markers are positioned on the *x*-axis based on the genomic order of the gene models in which the polymorphism was scored. The significance of the trait association, as -log10P values, plotted on the *y*-axis. The horizontal purple and cyan lines represent false discovery rate (FDR) threshold at 5% and the threshold for Bonferroni significance of 0.05, respectively. Chromosomes of *B. napus* are labelled A1–A10 and C1–C9, shown in alternating black and red colours to allow boundaries to be clearly distinguished. Dark opaque points are simple SNP markers (i.e. polymorphisms between resolved bases) and hemi-SNPs that have been directly linkage-mapped, both of which can be assigned to one genome, whereas light points are hemi-SNP markers (i.e. polymorphisms involving multiple bases called at the SNP position in one allele of the polymorphism) for which the genome of the polymorphism cannot be assigned.

As for GEM associations, reads per kb per million aligned reads (RPKM) were regressed against the trait, and R^2 and P values were calculated for each unigene. The gene models are positioned on the *x*-axis based on their genomic order, with the significance of the associated trait, as -log10P, plotted on the *y*-axis. The horizontal purple and cyan lines represent false discovery rate (FDR) threshold at 5% and the threshold for Bonferroni significance of 0.05, respectively.
