# Supplementary material for: Data in support of genetic architecture of glucosinolate variations in Brassica napus
Source: Data Brief. 2019 Aug 14;25:104402. doi: 10.1016/j.dib.2019.104402 (PMC6722234; doi:10.1016/j.dib.2019.104402)
Supplement: Supplementary file 1 [file mmc1.zip › Appendix7_QQLeaf.pdf]

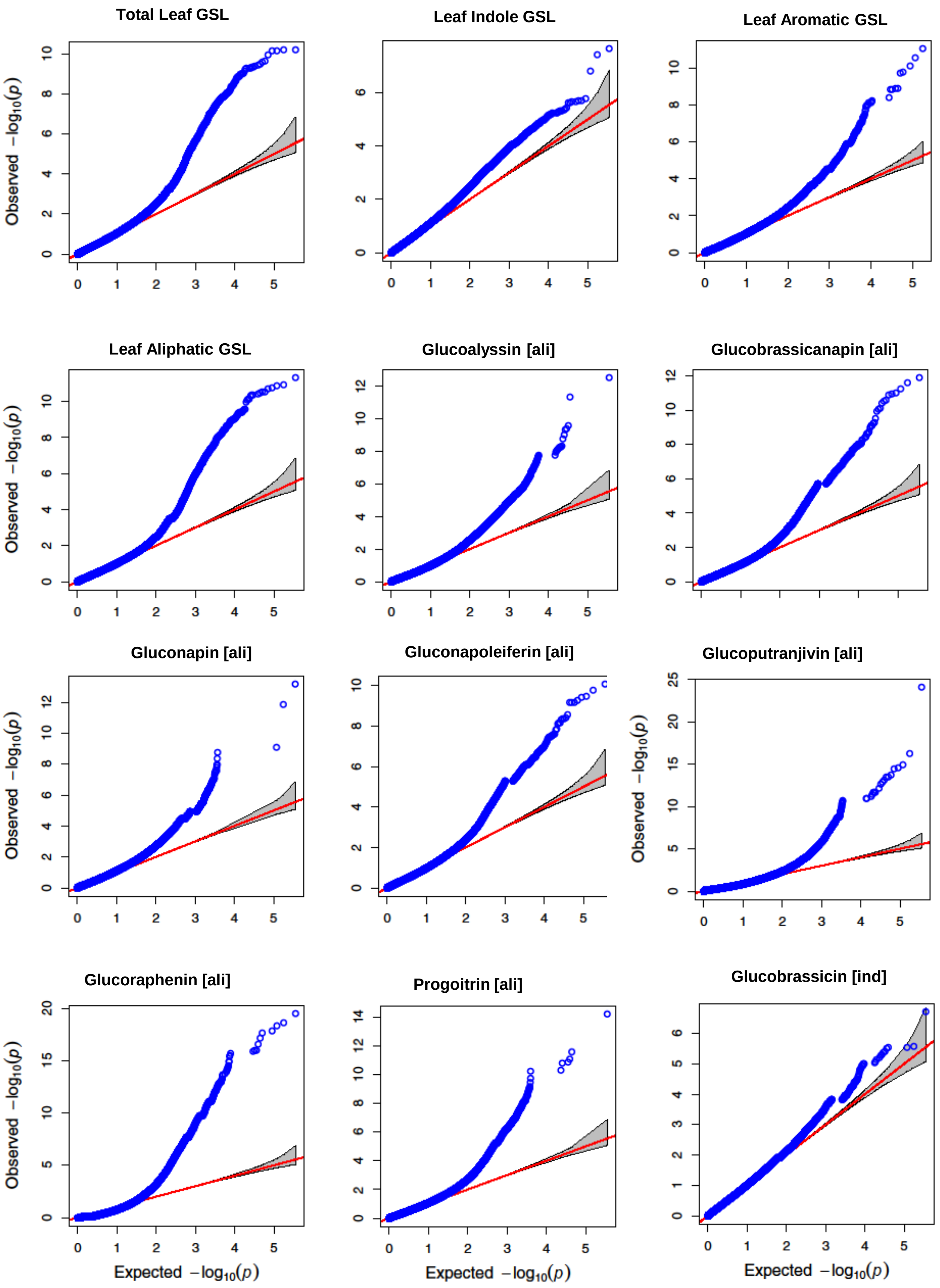

Appendix 7. QQ plots from SNP association analysis for leaf glucosinolates. The Y-axis is the observed- and the X-axis is expected observed negative base 10 logarithm of the *P*-values under the assumption that P-values follow a uniform distribution. The dotted lines show 95% confidence interval for the QQ-plot under the null hypothesis of no association between the SNP and the trait..

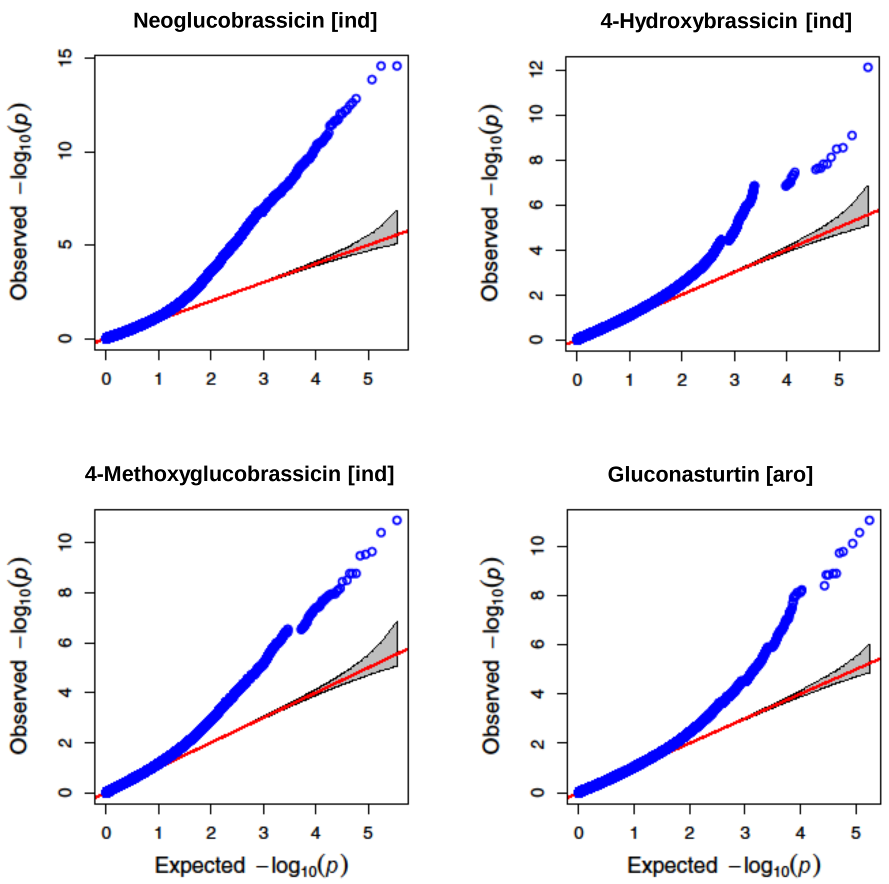

Appendix 7. QQ plots from SNP association analysis for leaf glucosinolates. The Y-axis is the observed- and the X-axis is expected observed negative base 10 logarithm of the *P*-values under the assumption that P-values follow a uniform distribution. The dotted lines show 95% confidence interval for the QQ-plot under the null hypothesis of no association between the SNP and the trait..
