# Supplementary figures and images for: Data in support of genetic architecture of glucosinolate variations in Brassica napus
Source: Data Brief. 2019 Aug 14;25:104402. doi: 10.1016/j.dib.2019.104402 (PMC6722234; doi:10.1016/j.dib.2019.104402)

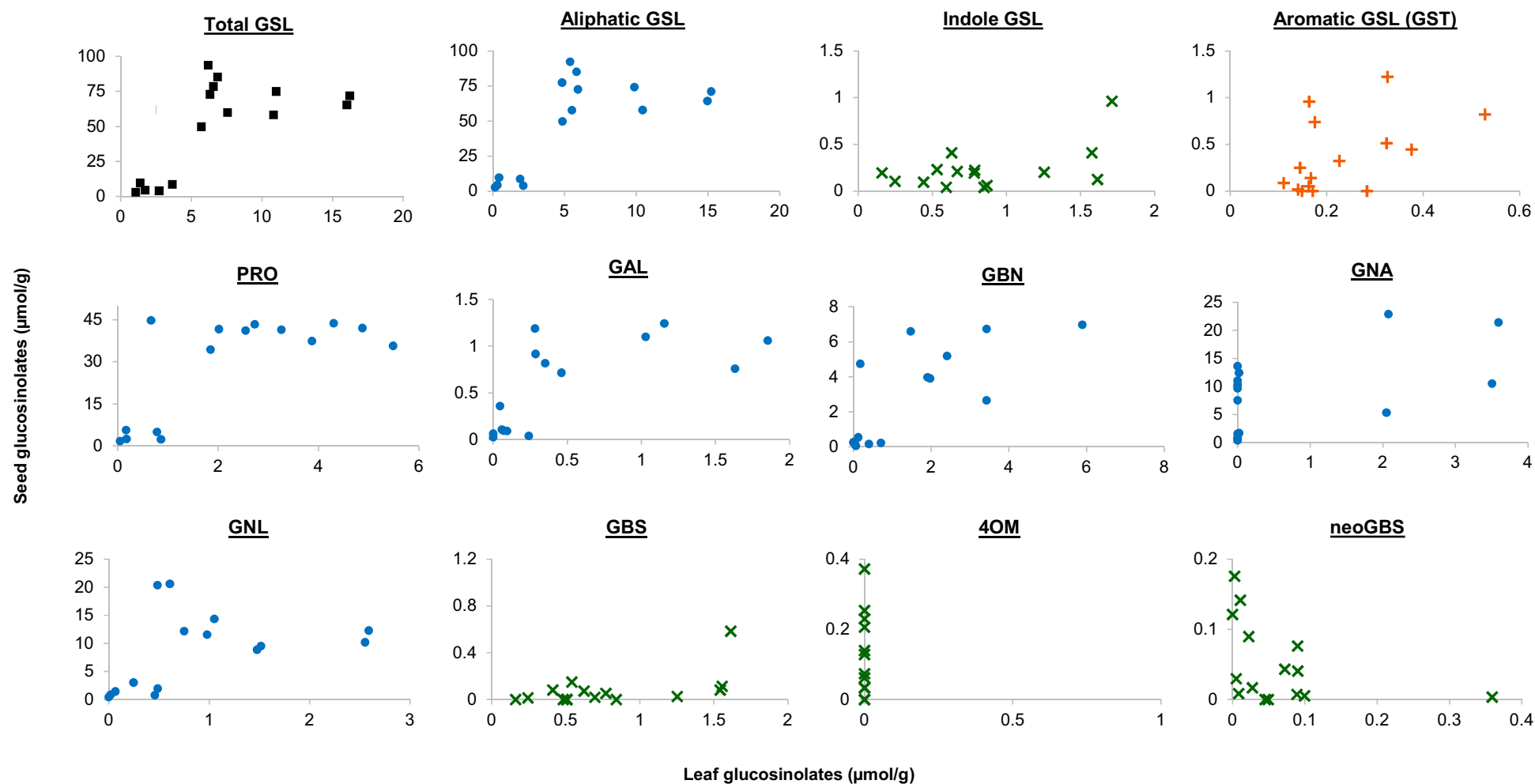

Supplement: Supplementary file 1 [file mmc1.zip › Appendix19_LeafSeedGSL_v2.pdf]
